# Prevalence of tooth wear diseases in patients with diabetes and its impact on the quality of life in Dakshina Kannada population: a cross sectional study

**DOI:** 10.12688/f1000research.140180.1

**Published:** 2023-10-26

**Authors:** Poojakumari Sinha, Sangeeta Nayak, Lavangi Sehgal, Ramya Shenoy Kudupi

**Affiliations:** 1 Department of Periodontology, Manipal College of Dental Sciences, Mangalore, Manipal Academy of Higher Education, Manipal, Karnataka, 575001, India; 2 Department of Public Health Dentistry, Manipal College of Dental Sciences, Mangalore, Manipal Academy of Higher Education, Manipal, Karnataka, India

**Keywords:** Attrition, Abrasion, Erosion, Tooth wear, Quality of life

## Abstract

**Background:**

Early detection and management of tooth wear (TW) has not been given due consideration in the dental profession. Thus, this study aimed to explore the prevalence of TW in adults with diabetes in Dakshina Kannada population using the Basic Erosive Wear Examination (BEWE) and recording its impact on quality of life using the Oral Health Impact Profile (OHIP-14).

**Methods:**

In total, 236 dentate adults with diabetes and TW, who visited the Manipal College of Dental Sciences, Mangalore were recruited to the study. Assessment of TW was done using BEWE during examination of each participant and was categorized as none, mild, moderate, and severe. Impact of TW on quality of life was assessed using the OHIP-14.

**Results:**

Overall, 81 (34.30%) individuals had a poor score on the basic erosive tooth index, 82 (34.70%) had a medium score, . The level of TW, both moderate and severe (high), affected quality of life. Erosion, abrasion and attrition were seen in a large proportion of the study population. The diabetic status of the individual was collected from their medical file. The HbA1C level was found to range from good, fair and poor control. The quality of life among the study population was affected because of TW and diabetes. Few participants had hypertension along with diabetes. The majority of study participants were on oral hypoglycaemic agents. Participants used tooth brushes and tooth paste and brushing frequency varied between once or twice daily.

**Conclusions:**

In the study population, there were moderate and severe TW patterns. Quality of life was found to be impacted by TW. Dental professionals must give proper consideration to the influence of TW on quality of life in addition to clinical findings. This will make it easier to offer preventive or restorative management depending on the situation.

## Introduction

People are keeping more natural teeth as they live longer lives. It is vital to have healthy, functional teeth when eating and following a regular diet. Tooth wear (TW), on the other hand, has a detrimental influence on the survival of natural teeth. TW, also known as tooth surface loss (TSL), has recently been identified as a serious oral health concern. ^1^^–^^3^ TW is an insidious, multifactorial disease that affects enamel and dentine, and it may endanger tooth survival and the quality of life linked with dental health among those who are affected. ^4^ Epidemiological studies support the hypothesis that TW is growing increasingly severe and frequent, not just among older adults but also among those approaching adulthood. ^5^^,^^6^ The three most often recognized aetiologies of TW are erosion, attrition, and abrasion. ^6^ Abfraction, the fourth aetiological factor, has been acknowledged by some but is not accepted by all. ^6^ These are associated with a variety of clinical symptoms. Given the aetiology's complexity, a definitive diagnosis may be difficult to achieve. TW influences both aesthetics and functionality.

Patients suffering with TW seek dental care as a result. The chemical breakdown of enamel or dentine produced by non-bacterial acids from dietary or gastric sources is known as erosion. The first stage is characterized by dull “silky glazed” enamel surfaces and enamel loss. The palatal surfaces of the maxillary anterior teeth are affected because the tongue and buccal mucosa shelter the other surfaces from exposure to stomach acid in the early stages of gastric erosion. ^6^ Dietary erosion is characterized by widespread cupped-out lesions, with the pattern being unique to the individual's habits and diet.

Attrition is a gradual deterioration of tooth structure caused by tooth-to-tooth contact in the absence of a foreign object. It often affects the incisal/occlusal region of the teeth. ^7^ Abrasion is the mechanical wear out of tooth structure that does not require tooth-to-tooth contact, a problem that commonly affects the cervical area of the teeth and is generally caused by poor tooth brushing practices that remove acid-weakened enamel and dentine. ^7^

Diabetes mellitus, one of the most common diseases on a regional and international scale, is a major cause of morbidity in most countries. ^8^^,^^9^ Diabetes is increasing in prevalence worldwide. According to data from the International Federation of Diabetes, 642 million people worldwide will have diabetes by 2040, up from 415 million in 2015. More than 60% of the worldwide population living with diabetes are in Asia, where the prevalence ranges from 3% to 47.3%. ^1^ Diabetes affects almost 1.3 billion people in India, which has the world's second-highest incidence. According to the International Diabetes Federation, 72.9 million individuals in India had diabetes in 2017.

Diabetes prevalence has risen rapidly and is expected to rise further as a result of the rapid and continuous socioeconomic upheaval in developing countries. Diabetes types 1 and 2 (T2DM) are becoming increasingly frequent. T2DM, the most common type of diabetes, has been the primary driver to the global rise in diabetes prevalence. ^10^ Diabetes increases the risk of oral disease both directly and indirectly. ^11^ Periodontal disease affects around one-third of individuals with diabetes. Periodontitis can cause tooth loss or sensitivity. ^11^ Different oral diseases or issues impact daily life in a variety of ways. There have not been many studies done on wasting diseases and how they affect people's quality of life. As a result, while assessing dental requirements, clinical and psychological factors must be considered.

Patients with diabetes make up the majority of those for whom dental practitioners utilize oral health prevention and promotion to achieve optimal oral and overall health. The incidence, severity, and underlying causes of tooth wear should be understood by dental practitioners. As a result, it is critical to research the frequency and severity of tooth wear among patients with diabetes, as well as how it impacts the quality of life of the general Dakshina Kannada community. Thus, the present research aimed to explore the prevalence of tooth wear in adults with diabetes in Dakshina Kannada population.

## Methods

From 29 ^th^ July 2022 to September 2022, this cross-sectional study was carried out at the Department of Periodontology, Manipal College of Dental Sciences Mangalore. The study protocol was submitted to the Manipal College of Dental Sciences Mangalore, Manipal Academy of Higher Education, Manipal, India's institutional ethics and review board (protocol no: 22023; approval date 28/07/22). Convenient sampling method was followed for the recruitment of the study participants. The patients who visited OPD of Manipal College of Dental Sciences, Mangalore during the study period were recruited in the study. Before joining the study, each study participant signed an informed consent form. Based on the inclusion and exclusion criteria, 236 people completed the clinical examination and questionnaire.

### Eligibility criteria

*Inclusion criteria*

Participants over the age of 18 years and above with a confirmed diagnosis of diabetes mellitus and willing to participate in the study were recruited for the research.

*Exclusion criteria*

Participants having a history of using pharmaceuticals for dental difficulties were excluded from the research, as were those with systemic diseases such as paralysis, leukaemia, epilepsy, depression, and other psychiatric ailments that impact TW, as well as those getting treatment for wasting diseases.

### Variables

Each participant's demographic information was collected using a pre-structured case form. Diabetes mellitus information, including type, duration, medication type, and any related diseases, was obtained using their medical file and a structured questionnaire, the case proforma can be found as *Extended data.* ^18^ Each participant was asked about their tooth-brushing regimen, including how long and how frequently they washed their teeth. We gathered data on diet and aerated beverage use.

We examined for evidence of tooth decay, gingivitis, periodontitis, missing teeth, sensitive teeth, bruxism, attrition, erosion, or abrasion during the clinical examination. The Basic Erosive Wear Examination (BEWE) was developed to track wasting and evaluate tooth deterioration. When there is no tooth wear, the first loss of surface tooth structure is 1, the distinct defect, hard tissue loss of 50% of the surface area is 2, and hard tissue loss of 50% of the surface area is 3. Dentine participation is seen in scores 2 and 3. Following the clinical evaluation, each participant was asked to complete the Oral Health Impact Profile (OHIP-14) questionnaire. The OHIP-14 ^12^ contains a questionnaire designed to examine the social consequences of disorders that may jeopardize oral health. Participants in the research were asked to describe any issues from the preceding year using the OHIP-14 rating scale. A high OHIP-14 scale score indicated a poor quality of life.

### Bias

Blinding of the investigators and participant was not required as the population only included individuals with diabetes. The reporting bias was taken care of by reporting actual results and confounding agents were excluded to prevent measurement bias.

### Study size

The sample size for the study was calculated using the following formula:

$$n={\left(\frac{Z_{\alpha /2}}{d}\right)}^{2}p\left(1-p\right)$$

Thus, a minimum of 197 participants were needed for the study.

### Statistical analysis

Statistical analysis was carried out using IBM SPSS Statistics (20.0) (IBM SPSS® statistics). It was necessary to tabulate the descriptive statistics. A proportional test was used to calculate frequency and distribution. Kruskal Wallis test was applied to check the association between BEWE of patients with diabetes with total OHIP scores. Mann-Whitney test was applied to check within the groups. The result showed statistical significance between the mild to severe BEWE group. The level of significance was set at p<0.05 and post Bonferroni correction it was p<0.02.

## Results

The current study included 236 individuals with diabetes in total. There were 171 men (72.50%) and 65 women (27.50%). In total, five (2.10%) of the participants were homemakers, whereas 53 (22.50%) worked in various occupations. A total of 108 (45.80%) were self-employed, while 65 (27.50%) were professionals ( Table 1). Overall, 14 (5.90%) of the study participants had diabetes and hypertension. A total of 136 (57.60%) of research participants used oral hypoglycaemic medication; 48 (20.30%) were using insulin, and 52 (22.00%) were taking ayurvedic drugs ( Table 1).

**Table 1.** Demographic details of study participants.

Details		Count	Column N %
Gender	Female	65	27.5%
	Male	171	72.5%
Occupation	Home Maker	5	2.1%
	Job	53	22.5%
	Business	108	45.8%
	Profession	65	27.5%
	Other	5	2.1%
Diabetes+ hypertension	Absent	222	94.1%
	Present	14	5.9%
Type of medication	Oral Hypoglycaemic	136	57.6%
	Insulin Preparation	48	20.3%
	Ayurvedic Medication	52	22.0%
	Homeopathic Medication	0	0.0%
Last HbA1C level	4-6 excellent	98	41.5%
	7-8 good	130	55.1%
	9-14 poor	8	3.4%
Diabetic complication	No	234	99.2%
	Yes	2	0.8%
Dental history	Absent	6	2.5%
	Present	230	97.5%
Decay	Absent	121	51.3%
	Present	115	48.7%
Gingivitis	Absent	118	50.0%
	Present	118	50.0%
Periodontitis	Absent	114	48.3%
	Present	122	51.7%
Hopeless tooth	Absent	233	98.7%
	Present	3	1.3%
Missing	Absent	38	16.1%
	Present	198	83.9%
Sensitivity	Absent	140	59.3%
	Present	96	40.7%
Bruxism	Absent	204	86.4%
	Present	32	13.6%
Any other	Absent	212	89.8%
	Present	24	10.2%
Diet	Veg	62	26.3%
	Non-Veg	40	16.9%
	Mixed	134	56.8%
Aerated drink intake	No	84	35.6%
	Yes	152	64.4%
	Daily	0	0.0%
	Weekly	0	0.0%
Aerated drink frequency	NA	84	35.6%
	Daily	2	0.8%
	Weekly	150	63.6%

**Figure 1.**
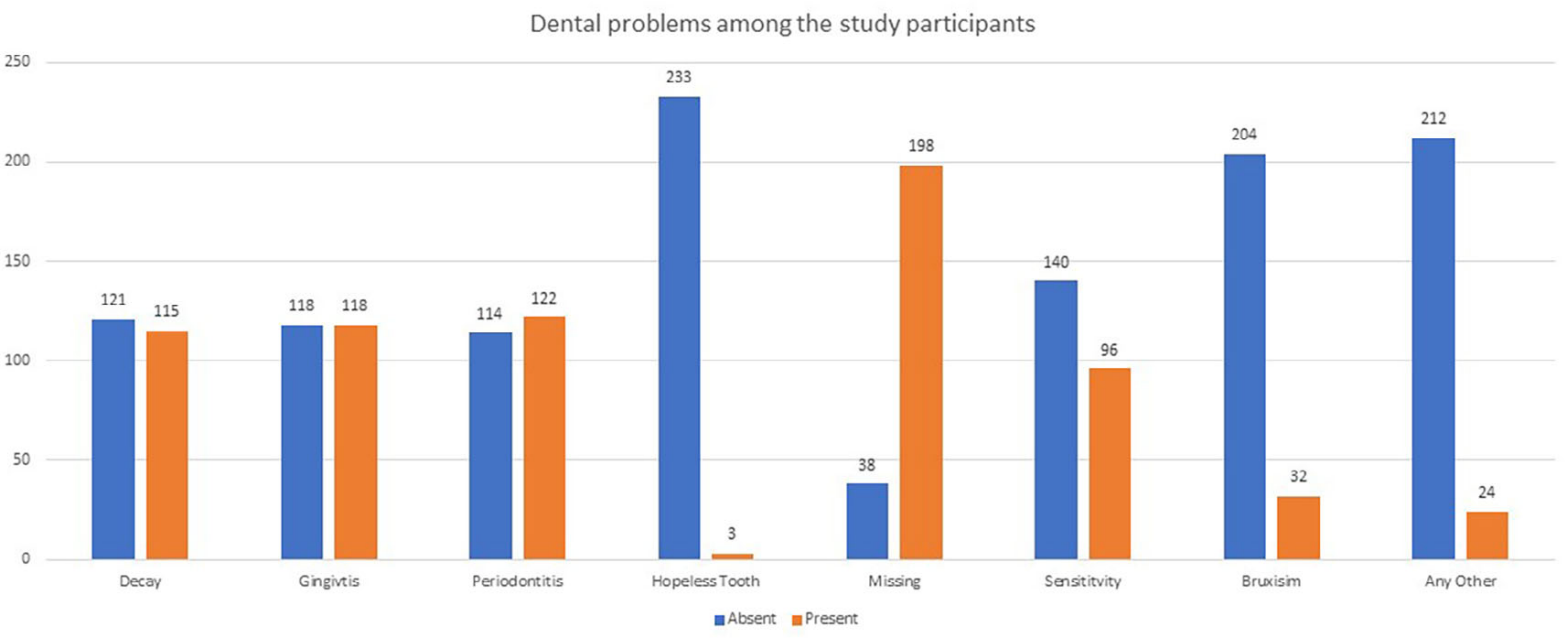
Various dental problems among the study participants.

In total, 98 (41.50%) individuals had excellent (4-6) previous HbA1C readings, 130 (55.10%) had good (7-8) levels, and eight (3.40%) had bad (9-14) levels ( Table 1). Overall six (2.50%) people had no dental concerns. Around 230 participants in the research had different oral conditions ( Figure 1). Around three (1.30%) of the 236 research participants had hopeless teeth or teeth with a poor prognosis. The bulk of the study population (134 (56.80%)) followed a mixed diet; 62 were vegetarians (26.30%). Among the research participants, 65 (27.50%) used horizontal strokes, while 134 (56.80%) used mixed strokes. A total of 229 (97.00%) of the research participants brushed their teeth twice every day for less than 2 minutes. Whereas seven (3.00%) cleaned their teeth twice every day for more than 2 minutes ( Table 2).

**Table 2.** Oral hygiene practice among study participants.

Details		Count	Column N %
Tooth brushing	Vertical	0	0.0%
	Horizontal	65	27.5%
	Mixed	171	72.5%
Brushing time	<2 minutes	229	97.0%
	>2 minutes	7	3.0%
Brushing Method	Nill	124	52.5%
	Mouthwash	6	2.5%
	Floss	53	22.5%
	Others	53	22.5%
Brushing material	Toothpaste and toothbrush	236	100.0%
	Powder	0	0.0%
	Others	0	0.0%
Brushing frequency	Once daily	107	45.3%
	Twice daily	129	54.7%

**Figure 2.**
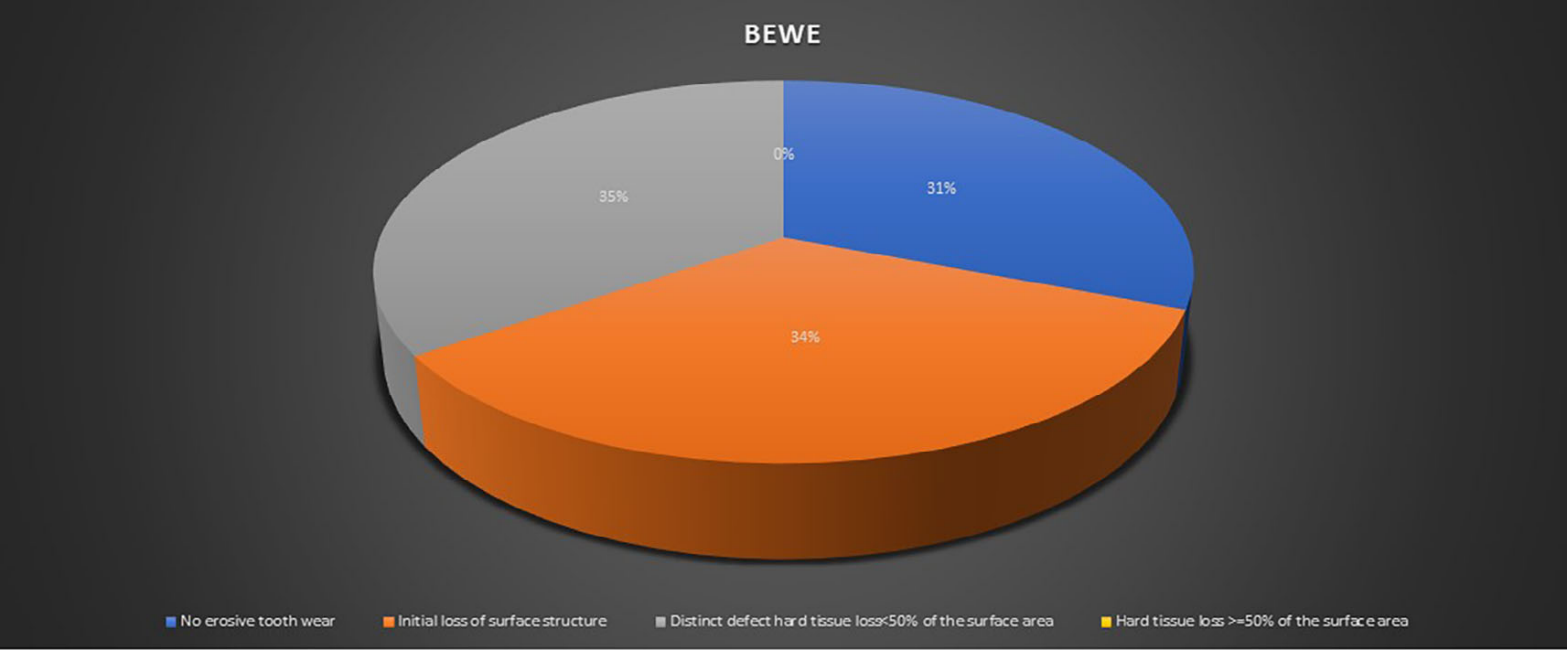
Basic Erosive Wear Examination (BEWE) score among study participants.

**Figure 3.**
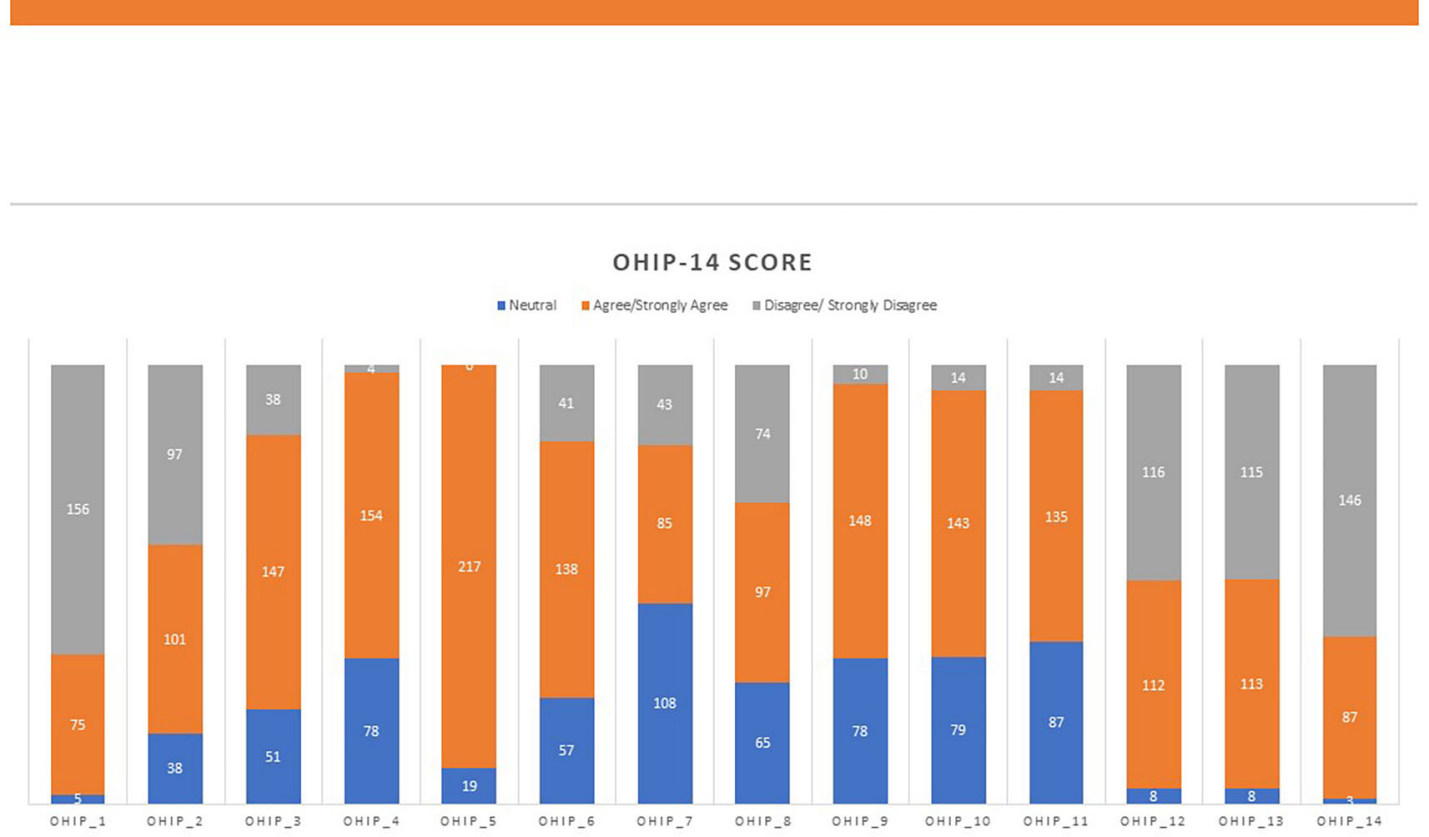
Oral Health Impact Profile (OHIP-14) score among study participants.

In total, 53 (22.50%) of the research participants used dental floss. Aerated drinks were consumed by 152 people (64.40%). Aerated drinks were consumed on a weekly basis by 150 people (63.60%). Attrition, abrasion, and erosion are examples of wasting diseases; 223 (94.50%) of the study participants had different types of wasting diseases. Erosion was seen in 228 cases (96.60%), whereas abrasion was observed in 221 cases (93.60%) ( Table 3 and Figure 2). The presence of wasting diseases and the severity was recorded in the oral examination. The participants gained awareness regarding the wasting diseases by their dentists or through newspapers, and magazines. Approximately 96 (40.70%) of the research participants reported sensitivity to hot and cold meals. A total of 96 (40.70%) of the participants reported sensitivity as a result of their teeth becoming thin, short, and structurally damaged. Around 32 (13.60%) of the research participants would grind their teeth. The majority of the participants last went to the dentist one or two years ago. Overall, 129 research participants (54.70%) brushed their teeth twice every day ( Table 2). The fundamental erosive tooth index was seen in 73 (30.90%) of the cases. There was an early loss of surface tooth structure in 81 (34.30%), and in 82 (34.70%) participants ( Table 3). Each participant thoroughly filled out the questionnaire regarding their quality of life ( Table 4 and Figure 3). Chewing food was challenging for 147 (91.90%) of the subjects. Responses were grouped into three sections (neutral, agree/strongly agree, disagree/strongly disagree) ( Figure 3 and Table 4). The association between tooth wear and diabetes and its impact on quality of life was assessed. The result showed statistical significance between the mild to severe BEWE group ( Table 5).

**Table 3.** Tooth wear and Basic Erosive Wear Examination score among study participants.

Details		Count	Column N %
Attrition	Absent	13	5.5%
	Present	223	94.5%
Abrasion	Absent	15	6.4%
	Present	221	93.6%
Erosion	Absent	8	3.4%
	Present	228	96.6%
BEWE	No erosive tooth wear	73	30.9%
	Initial loss of surface structure	81	34.3%
	Distinct defect hard tissue loss<50% of the surface area	82	34.7%
	Hard tissue loss > = 50% of the surface area	0	0.0%

**Table 4.** Oral Health Impact Profile (OHIP-14) responses by the study participants.

Details		Count	Column N %
OHIP_1	Neutral	5	2.1%
	Agree/Strongly Agree	75	31.8%
	Disagree/Strongly Disagree	156	66.1%
OHIP_2	Neutral	38	16.1%
	Agree/Strongly Agree	101	42.8%
	Disagree/Strongly Disagree	97	41.1%
OHIP_3	Neutral	51	21.6%
	Agree/Strongly Agree	147	62.3%
	Disagree/Strongly Disagree	38	16.1%
OHIP_4	Neutral	78	33.1%
	Agree/Strongly Agree	154	65.3%
	Disagree/Strongly Disagree	4	1.7%
OHIP_5	Neutral	19	8.1%
	Agree/Strongly Agree	217	91.9%
	Disagree/Strongly Disagree	0	0.0%
OHIP_6	Neutral	57	24.2%
	Agree/Strongly Agree	138	58.5%
	Disagree/Strongly Disagree	41	17.4%
OHIP_7	Neutral	108	45.8%
	Agree/Strongly Agree	85	36.0%
	Disagree/Strongly Disagree	43	18.2%
OHIP_8	Neutral	65	27.5%
	Agree/Strongly Agree	97	41.1%
	Disagree/Strongly Disagree	74	31.4%
OHIP_9	Neutral	78	33.1%
	Agree/Strongly Agree	148	62.7%
	Disagree/Strongly Disagree	10	4.2%
OHIP_10	Neutral	79	33.5%
	Agree/Strongly Agree	143	60.6%
	Disagree/Strongly Disagree	14	5.9%
OHIP_11	Neutral	87	36.9%
	Agree/Strongly Agree	135	57.2%
	Disagree/Strongly Disagree	14	5.9%
OHIP_12	Neutral	8	3.4%
	Agree/Strongly Agree	112	47.5%
	Disagree/Strongly Disagree	116	49.2%
OHIP_13	Neutral	8	3.4%
	Agree/Strongly Agree	113	47.9%
	Disagree/Strongly Disagree	115	48.7%
OHIP_14	Neutral	3	1.3%
	Agree/Strongly Agree	87	36.9%
	Disagree/Strongly Disagree	146	61.9%

**Table 5.** Association between tooth wear and its impact on quality of life.

Group	Comparison group	Test value	Significance (p<0.02) – Bonferroni correction
Mild	Moderate	2723.500	0.393
	Severe	1266.500	**0.000**
Moderate	Severe	2828.000	0.084

## Discussion

The current study looked at the prevalence of tooth wear and its influence on the quality of life of those with diabetes. Responses were received from male and female participants ranging in age from 18 to 80 years old, as in previous investigations. ^1^^,^^4^ According to Bartlett e *t al.*, more male patients than female patients are sent to the dentist with concerns of TW. ^7^ This was consistent with the findings of the current study. ^1^^,^^7^ There is no evidence that men and women have distinct tooth forms or saliva compositions. The reasons of TW are numerous.

A study by Srisilapanan *et al.* (2018), like ours, covered participants with type 2 diabetes between the ages of 35 and 74 years old. Patients with diabetes had a higher incidence of tooth loss, which is consistent with previous research. ^1^ According to data from the American Dental Association, ^11^ diabetes is responsible for one out of every five tooth loss cases. According to the most current research, the most common kind of TW was attrition, which is consistent with previous findings. ^1^ Study participants in the present study had a greater rate of TW, which was consistent with previous research. ^1^ The severity of minor TW and age were shown to be substantially associated.

Diabetes and periodontitis are two exceedingly frequent chronic, noncommunicable diseases that pose severe public health problems for the worldwide population. For a long time, dental specialists have recognized that these two diseases are linked. Periodontitis is known as the sixth complication of diabetes. Diabetes raises the risk of periodontitis by increasing inflammation in periodontal tissues. ^13^ Participants in the research had gingivitis, periodontitis, decay, and other dental disorders. The inability to maintain excellent oral hygiene may be related to the challenges (sensitivity and discomfort) caused by wasting disorders.

Diabetes and periodontal disease treatment need a lifelong strategy tailored to the patient's individual circumstances. The majority of survey participants used mixed and horizontal strokes to clean their teeth. This must be corrected because it is a common occurrence and a substantial factor to wasting sickness. Any oral health promotion program must include tooth brushing instruction since it improves people's oral health knowledge, attitudes, and habits. Individuals may use it to construct customised dental care programs for themselves. The research participants had moderate to high scores on the basic erosive tooth index. The study looked at how tooth decay affects people's quality of life. Patients who had moderate to severe TW had a worse quality of life. If monitoring TW is made required at every clinical examination, it is less likely that it will be neglected. Every visit should include a TW evaluation to reduce the likelihood of patients’ early warning signs going ignored and no preventative steps being implemented. Dental practitioners should pay attention to their patients’ clinical features and how they affect their patients’ quality of life. On the basis of demand, specialized, preventative, or rehabilitative treatment should be made available. OHIP-14 can be used to quantify the impact of oral diseases on important elements of daily living. ^3^ Traditional or professionally developed assessment methods for evaluating tooth wear may be utilized to prioritize treatment for individuals who require it the most urgently. ^14^^,^^15^

Further study is needed to discover how each of these clinical characteristics influences quality of life. By doing so, it may be feasible to identify the elements that have the greatest impact on people's lives. Future research should look at the link between dental hypersensitivity, aesthetics, tooth deterioration, and quality of life.

Several strategies may be used to assess the severity of various oral disorders in the population. They are often used in the community to minimize the occurrence of diseases and other ailments. The current study used an instrument such as the OHIP-14 to assess the prevalence of TW and its impact on well-being in diabetes. This is supported by other material that is currently available. ^16^^,^^17^

## Conclusions

In conclusion, the findings of this study provide evidence for the consequences of dental wear disorders in patients with diabetes and their implications on the standard of life of adults in the Dakshina Kannada community. Setting a goal and combining planning and self-monitoring in specific instructions will help you mark the behaviour change more successfully. These people may benefit from regular dental checkups and rigorous adherence to oral hygiene regulations. Future studies should concentrate on this topic.

## Data Availability

Figshare: Tooth wear in diabetic population, https://doi.org/10.6084/m9.figshare.23635173. ^18^ This project contains the following underlying data:
- Datasheet.xlsx - data-TW.xlsx Figshare: Tooth wear in diabetic population, https://doi.org/10.6084/m9.figshare.23635173. ^18^ This project contains the following extended data:
- case proforma.docx Data are available under the terms of the Creative Commons Zero “No rights reserved” data waiver (CC0 1.0 Public domain dedication).
