# Supplementary material for: Prevalence of tooth wear diseases in patients with diabetes and its impact on the quality of life in Dakshina Kannada population: a cross sectional study
Source: F1000Res. 2023 Oct 26;12:1411. [Version 1] doi: 10.12688/f1000research.140180.1 (PMC11320185; doi:10.12688/f1000research.140180.1)
Supplement: Peer review history

# Reviewer report: Reviewer response for version 1

**DOI:** 10.5256/f1000research.153511.r306570

**Published:** 2024-08-12

**Authors:** Sabin Siddique

**Affiliations:** 1 Department of Public Health Dentistry, MES Dental College and Hospital, Perinthalamanna, Kerala, Italy

It is a relevant study and its impact on quality of life of diabetic patients , as it is one of the frequently reported lifestyle disease in the country and wasting diseases based research  is rarely reported among diabetic patients so it is important for dentists to understand the risk of diabetes on oral health.

Is the work clearly and accurately presented and does it cite the current literature?

Yes

If applicable, is the statistical analysis and its interpretation appropriate?

Yes

Are all the source data underlying the results available to ensure full reproducibility?

Yes

Is the study design appropriate and is the work technically sound?

Yes

Are the conclusions drawn adequately supported by the results?

Yes

Are sufficient details of methods and analysis provided to allow replication by others?

Yes

Reviewer Expertise:

dental public health

I confirm that I have read this submission and believe that I have an appropriate level of expertise to confirm that it is of an acceptable scientific standard.

# Reviewer report: Reviewer response for version 1

**DOI:** 10.5256/f1000research.153511.r218390

**Published:** 2024-04-12

**Authors:** Menaka Karalwad

**Affiliations:** 1 Periodontics, Kaher, KLE VK Institute of Dental Sciences, Belagavi, Karnataka, India

A well written paper addressing the most common clinical conditions related to dental health. The study would be justified if authors can comment regarding selection of diabetic patients only as their sample and explain the influence of diabetes on tooth wear when compared to systemic healthy individuals otherwise. The study design can be described as descriptive cross-sectional single center study. In methodology the details on who performed the clinical examination-single/multiple examiners to avoid bias can be stated. Habit history of bruxism needs to be mentioned as its important in tooth wear. The age group 18-80 years, it would be interesting to know & explore the association between age groups for the main outcomes assessed.

Is the work clearly and accurately presented and does it cite the current literature?

Yes

If applicable, is the statistical analysis and its interpretation appropriate?

Yes

Are all the source data underlying the results available to ensure full reproducibility?

Yes

Is the study design appropriate and is the work technically sound?

Partly

Are the conclusions drawn adequately supported by the results?

Yes

Are sufficient details of methods and analysis provided to allow replication by others?

Yes

Reviewer Expertise:

Periodontal Medicine, Clinical Periodontics, Lasers in Periodontics, Periodontal Microbiology

I confirm that I have read this submission and believe that I have an appropriate level of expertise to confirm that it is of an acceptable scientific standard.
